# Practitioner's Bookshelf

**Published:** 1892-02-27

**Authors:** 


### PRACTITIONERS' BOOKSHELF.

### YELLOW FEVER.*

Cases of yellow fever occur so rarely in England that the majority of practitioners at home have never seen a case, and consequently the English literature of the subject is not very satisfactory. But during the last few years a number of able contributions by practitioners who have had ample experience have done much towards rendering our knowledge more exact and up to date.

The little book before us is based upon a paper read by the author before the Royal Medical Society of Edinburgh some years ago.

Dr. Martin refers to the presence of micro-organisms observed by Cornil and Babès. They found chains of diplococci in the capillaries of various organs in the case of yellow fever. Later on, however, Babès found in two cases short, rod-like bacilli in the mucous membrane of the small intestine, very similar to the typhoid bacilli.

Domingo Freire regards a micro-organism, which he has named crypto-coccus xanthogenicus, or micrococcus amaril, as the cause of yellow fever; but Flügge says that his observations are based upon errors. According to Duncan, who has done good service by his investigation of yellow fever, and from whose work Dr. Martin quotes as follows: "In 1883 Freire reported to the Biological Society of Paris that he had found a specific microbe in cases of yellow fever, and that he had proved his point by experimenting on guinea pigs. He also ascertained that the microbe secretes an alkaloid, which, when attenuated, can be used for preventive inoculation. These organisms Freire has also found in the soil in districts where yellow fever is prevalent. From December 22nd, 1884, to March 22nd, 1885, or a period of three months, 1,109 persons of different nationalities, whose ages ranged from one month to sixty years, have been inoculated in the deltoid region. Not one single case was severely attacked, and the very few who had suffered from the fever had it in the mildest form possible. In many cases the process was per-

*Yellow Fever: a Monograph. By James Martin, M.D. Pp. 56, with coloured map and charts. (E. and S. Livingston, Edinburgh.)

formed in houses where a few hours before death had taken place from yellow fever." And also the results in December, 1885, and January and February, 1886, as follows: "Among 3,051 subjects inoculated in Rio de Janeiro there were no deaths, whereas in the same localities and houses 278 unprotected individuals died of the disease. Lastly, Dr. Martin found in Rio de Janeiro the mortality to be 1·6 per cent. amongst those inoculated, and 13·7 per cent. amongst those not protected." Drs. Girand and Carwona use inoculations as advised by Freire, and with good results; and when we know that the usual mortality is at least 50 per cent. we cannot fail to appreciate Dr. Freire's labours in curbing this fatal disease, and urging the general introduction of inoculation.

As the outcome of a practical experience of this tropical scourge, it cannot fail to be an acquisition. Without adding much that is new on the subject, Dr. Martin reviews and analyses the results of many observers. A number of cases are briefly reported in the appendix; these will help one to realise the condition which is met with in yellow fever patients.

### THE "JOURNAL OF NERVOUS AND MENTAL DISEASE."

The first monthly number of this neurological journal, which is edited by Dr. C. H. Brown, of New York, with the active co-operation of twenty distinguished American neurologists, contains a number of contributions of value. Dr. H. A. Tomlinson writes on the indications for, and application of, physical culture in the treatment of insanity and allied diseases. His remarks are practical, and the drift of the article may be estimated from the following paragraph: "As a summary of what has been here stated, it seems to me safe to say that physical culture, systematically and progressively applied, is one of the best means at our command for the improvement of nutrition and the furthering of elimination, and that by its methods and persistence in tends to check irregular manifestations of nervous energy, and to guide them into normal channels of activity. I include under the head of physical culture, massage, passive and resistive muscular movements, galvanic and faradic muscular stimulations, systematic voluntary muscular movements, and light gymnastics —so called." Dr. Dercum writes on a case of railway spine, and a good article from the pen of Dr. Graeme Hammond extols the bicycle as a means of treatment of certain nerve diseases. Drs. C. L. Dana, David Nigter, and others contribute in this number. The "Neurological Digest" reviews contemporary neurological literature.

